# p27 Deficiency Cooperates with Bcl-2 but Not Bax to Promote T-Cell Lymphoma

**DOI:** 10.1371/journal.pone.0001911

**Published:** 2008-04-02

**Authors:** Ningli Cheng, Christopher I. van de Wetering, C. Michael Knudson

**Affiliations:** Department of Pathology, Program in Molecular and Cellular Biology, University of Iowa Roy J. and Lucille P. Carver College of Medicine, Iowa City, Iowa, United States of America; University of Helsinki, Finland

## Abstract

The effect of Bcl-2 on oncogenesis is complex and expression may either delay or accelerate oncogenesis. The pro-oncogenic activity is attributed to its well characterized anti-apoptotic function while the anti-oncogenic function has been attributed to its inhibition of cellular proliferation. Recent studies demonstrate that p27 may mediate the effects of Bcl-2 on cellular proliferation. We hypothesized that p27 may suppress tumor formation by Bcl-2 family members. To test this hypothesis, cell cycle inhibition and lymphoma development were examined in Lck-Bcl-2 and Lck-Bax38/1 transgenic mice deficient in p27. Strikingly, p27 deficiency synergistically cooperates with Bcl-2 to increase T cell hyperplasia and development of spontaneous T cell lymphomas. Within 1 year, >90% of these mice had developed thymic T cell lymphomas. This high penetrance contrasts with a one year incidence of <5% of thymic lymphoma in Lck-Bcl-2 or p27 −/− mice alone. In contrast, p27 deficiency had no effect on tumor formation in Lck-Bax38/1 transgenic mice, another model of T cell lymphoma. Histologically the lymphomas in p27 −/− Lck-Bcl-2 mice are lymphoblastic and frequently involve multiple organs suggesting an aggressive phenotype. Interestingly, in mature splenic T cells, Bcl-2 largely retains its anti-proliferative function even in the absence of p27. T cells from p27 −/− Lck-Bcl-2 mice show delayed kinetics of CDK2 Thr-160 phosphorylation. This delay is associated with a delay in the up regulation of both Cyclin D2 and D3. These data demonstrate a complex relationship between the Bcl-2 family, cellular proliferation, and oncogenesis and demonstrate that p27 up-regulation is not singularly important in the proliferative delay observed in T cells expressing Bcl-2 family members. Nonetheless, the results indicate that p27 is a critical tumor suppressor in the context of Bcl-2 expression.

## Introduction

The Bcl-2 oncogene was discovered at the t(14;18) translocation breakpoint in human B cell follicular lymphomas [1]–[3]. Bcl-2 is a member of a large family of proteins that are critical regulators of cell death. This family is comprised of both anti-apoptotic (Bcl-2, Bcl-X_L_) and pro-apoptotic members (Bax, Bak) [4], [5]. Bcl-2 predominantly resides at the outer mitochondrial membrane and is able to block a number of mitochondrial changes that occur with apoptosis such as loss of mitochondrial membrane potential (ΔΨm), release of mitochondrial proteins such as cytochrome C and Apoptosis-Inducing Factor (AIF), and opening of the mitochondrial permeability transition pore (PTP) [5], [6]. Bcl-2 also functions by preventing the formation of a complex that involves pro-apoptotic family members such as Bak and Bax [7]. Due to its anti-apoptotic function, Bcl-2 is thought to enhance oncogenesis by prolonging the survival of cells with pre-tumorigenic lesions and therefore increase the likelihood of malignancy [8].

Paradoxically, Bcl-2 expression is not always correlated with accelerated oncogenesis and a poor clinical outcome. In studies of human colon cancer [9]–[13] and breast cancer [14] increased Bcl-2 expression correlated with prolonged survival. In mouse models of cancer, Bcl-2 expression resulted in delayed tumor development in c-myc driven hepatocellular carcinoma [15], dimethylbenz(a)anthracene induced mammary tumors [16], and UV or chemical induced squamous cell carcinomas [17]. Moreover, we recently demonstrated that Bcl-2 suppressed lymphoma formation and chromosomal instability in transgenic mice expressing Bax [18], [19]. In many of these studies, the tumor-suppressive effect of Bcl-2 correlated with decreased cellular proliferation.

A variety of studies have shown Bcl-2 has a significant anti-proliferative effect on different cell types. In BAF3 cells following IL-3 withdrawal, Bcl-2 expressing cells rapidly arrested in the G1 phase of the cell cycle and were refractory to re-stimulation when IL-3 was added back [20]. Moreover, in both fibroblasts and IL-3 dependent cells arrested in G0, Bcl-2 expression increased the time to reach S phase [21]. Bcl-2 was also shown to accelerate cell cycle withdrawal into G0 [22]. Together, these findings demonstrate that Bcl-2 regulates cellular proliferation by controlling the G0 to G1 transition. Several studies on the effect of Bcl-2 on resting lymphocytes are consistent with this interpretation. B-lymphocytes isolated from Bcl-2 transgenic mice were resistant to activation and proliferation *in vitro* [23]–[25]. Similarly, T cells isolated from Bcl-2 transgenic, wild-type, and deficient mice demonstrated an inverse correlation between Bcl-2 levels and proliferation [26]. Furthermore, *in vivo*, B-lymphocytes from Bcl-2 transgenic mice displayed delayed activation following immunization [27].

An important point that remains controversial is whether the same or distinct functions of Bcl-2 account for the anti-proliferative and anti-apoptotic activities. In one study using cell lines, a single point mutation at tyrosine 28 (to Ala, Ser, and Phe) in the BH4 domain was able to ablate the anti-proliferative effect without affecting the anti-apoptotic function of Bcl-2 [21]. In contrast to this finding, other reports using this same mutation in cell lines and in transgenic mice demonstrated a tight correlation between the anti-apoptotic and anti-proliferative functions of Bcl-2 and demonstrated these two functions could not be separated [28], [29]. Alternatively, these reports suggest that cell cycle delay by Bcl-2 depends on the prolonged *in vivo* survival of these cells which results in reduced cell size and delayed cellular proliferation [29].

In multiple reports, molecular analysis of Bcl-2-mediated inhibition of cell cycle has consistently shown that p27 protein levels are elevated in cells expressing Bcl-2 [28]–[30]. P27 is a Cyclin Dependent Kinase Inhibitor (CKI) and a member of the Cip/Kip family [31]. In particular, p27 has been suggested to act by inhibiting the activation of CDK2, a critical G1 kinase [31]. While the mechanism by which Bcl-2 elevates p27 levels is unknown, one study reported that Bcl-2 was unable to inhibit cellular proliferation in p27 deficient cells [30]. These studies prompted us to examine the role of p27 in both cell cycle control and malignant transformation in mice over-expressing Bcl-2.

Here we report a striking synergistic cooperation between p27 deficiency and Bcl-2 in thymic lymphoma development. Despite this synergy, Bcl-2 largely retains its anti-proliferative function in p27 −/− deficient splenic T cells from mice prior to lymphoma development. These findings demonstrate a complex relationship between p27 and Bcl-2 with regards to cellular proliferation, and oncogenesis. The data show that up-regulation of p27 is not singularly important in the proliferative delay observed in T cells expressing Bcl-2. However, p27 functions as a critical tumor suppressor in the context of enforced Bcl-2 expression in thymocytes.

## Results

### T cell hyperplasia in p27 −/− Lck-Bcl-2 mice

**Figure 1:**
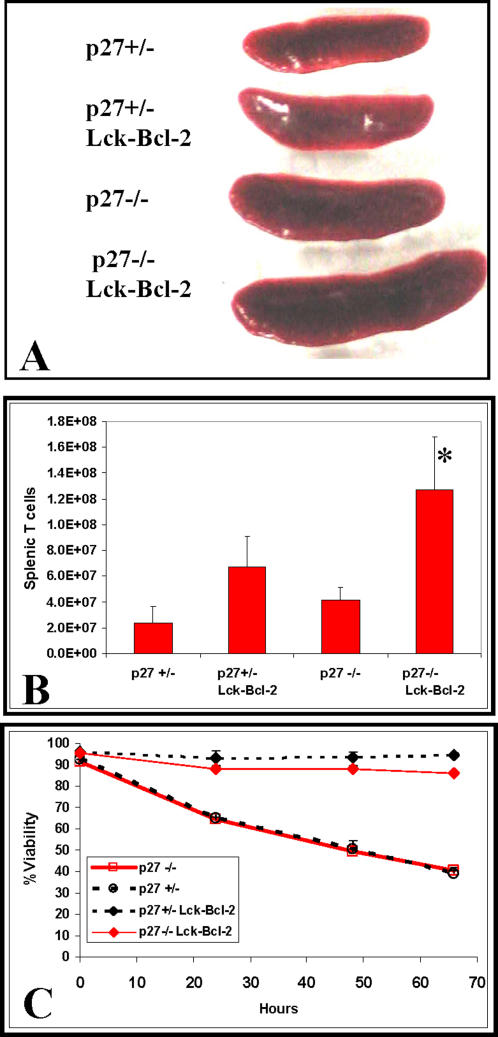
Lymphoid hyperplasia in p27 −/− Lck-Bcl-2 mice. Spleens were dissected and T cells isolated from p27 −/− Lck-Bcl-2 transgenic mice and analyzed for viability and total splenic T cells as described in the Materials and Methods. Control groups included p27 −/−, p27 +/−, and p27 +/− Lck-Bcl-2 mice. A) Representative images of spleens from mice of the indicated genotypes. B) Total splenic T cells were determined by staining with an anti-CD3 antibody as described in the Materials and Methods. The Mean±SD of at least three mice is shown for the genotypes indicated. * P value <0.01 compared to all three other groups. C) Purified T cells from mice of the indicated genotypes were prepared and cultured as described in the Materials and Methods. Viability was determined over time by using the Guava Flow cytometer and Viacount reagent. The mean±SD for duplicate samples are shown.

**Figure 2:**
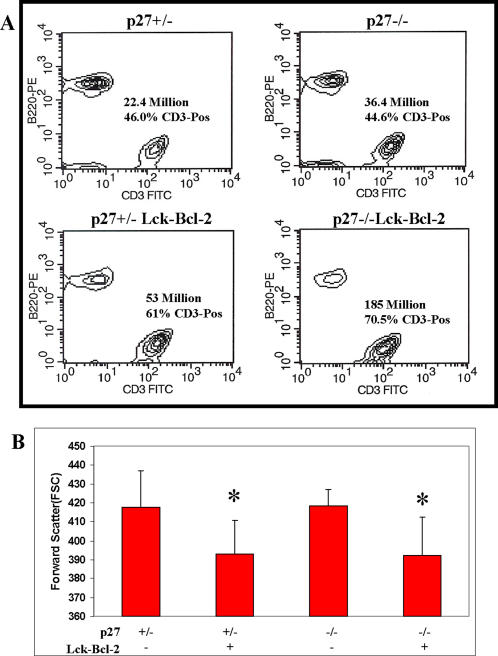
Splenic T cell hyperplasia and T cell size in p27 −/− Lck-Bcl-2 mice. A) Total splenic cells from mice of the indicated genotypes were isolated and stained with anti-B220-PE and anti-CD3-FITC antibodies as described in the Materials and Methods. The percentage of CD3-positive cells and the total number of splenic T cells (total # of splenic cells X %CD3) is shown. The data are representative of at least three mice from each genotype. B) The Mean Forward Scatter of CD3 positive splenic T cells was determined by staining with an anti-CD3 antibody as described in the Materials and Methods. The Mean±SD of at least 5 mice is shown for the genotypes indicated. * P<0.01 versus both the p27 +/− and p27 −/− mice using the unpaired Students T test. The p27 +/− Lck-Bcl-2 mice were not significantly different than the P27 −/− Lck-Bcl-2 mice (P = 0.89).

To examine whether p27 functions as a critical suppressor of cell proliferation, lymphoid hyperplasia, and oncogenesis downstream of Bcl-2, p27 deficient mice were crossed to mice over-expressing Bcl-2 in T cells (Lck-Bcl-2) [32]. Lymphoid hyperplasia was assessed by determining the number of T cells (CD3 +) and B cells (B220 +) present in the spleen [33]. As expected from previous studies, both Lck-Bcl-2 and p27 −/− mice had modestly enlarged spleens and increased splenic T cells. However, the combination of Lck-Bcl-2 and p27 −/− deficiency proved synergistic as the spleens were markedly enlarged (Figure 1A) and the number of splenic T cells dramatically increased (Figure 1B). As expected, Bcl-2 markedly prolonged *in vitro* T cell survival while p27 expression had no effect on survival with or without Lck-Bcl-2 (Figure 1C). Staining of splenic B and T cells demonstrates no change in the percentage of T cells in p27 −/− mice versus the p27 +/− control. In contrast, Bcl-2 expression markedly increased the percentage of T cells with p27 −/− mice showing an even higher percentage than the p27 +/− mice (Figure 2A). Previous results had demonstrated that Bcl-2 decreases T cell size [26] which was correlated with inhibition of cell cycle [29]. We therefore examined whether the reduction in splenic T cell size as measured by forward scatter was dependent upon p27. Bcl-2 effectively decreased splenic T cell size in p27 +/− and p27 −/− strains (Figure 2B).

### Developmental regulation of thymocyte proliferation in p27 −/− mice

**Figure 3:**
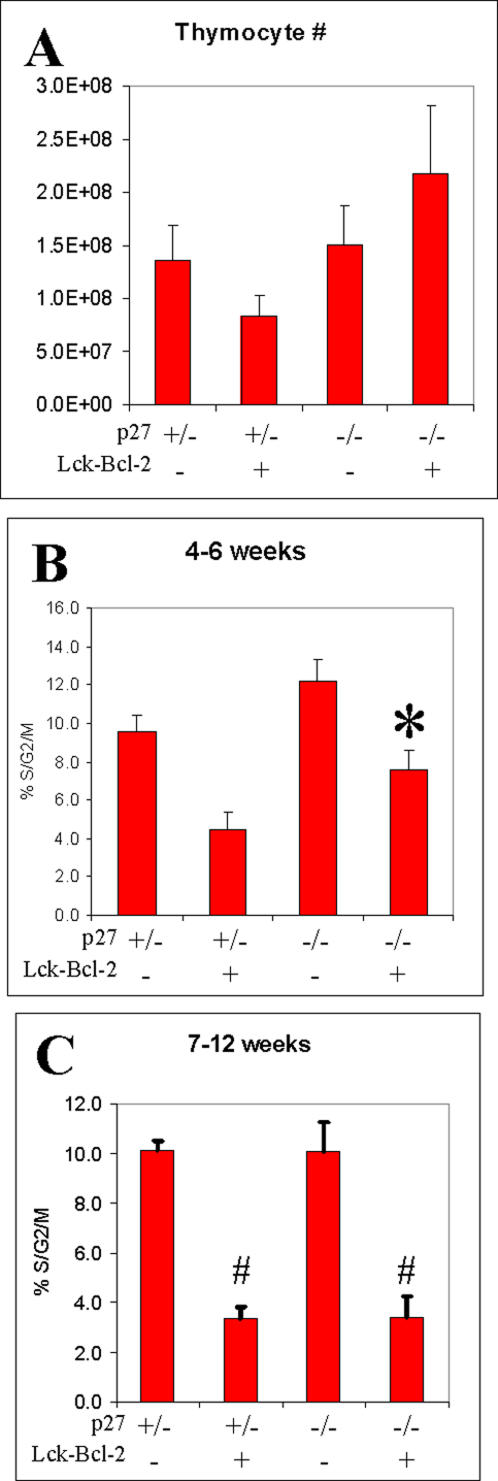
Thymocyte cellularity and cell cycle in p27 −/− Lck-Bcl-2 mice. A) Total thymocyte cellularity from mice of the indicated genotypes (Mean +/− SEM) between 4 and 12 weeks of age is shown. B) DNA content analysis was performed on freshly isolated thymocytes on mice of the indicated genotypes between 4 and 6 weeks of age. The mean and SD of at least 3 mice per group is shown. * P<0.01 versus the control p27 +/−, p27+/− Lck-Bcl-2 and p27 −/−mice using the unpaired Students T test. C) DNA content analysis was performed on freshly isolated thymocytes on mice of the indicated genotypes between 7 and 12 weeks of age. The mean and SD of at least 3 mice per group is shown. # P<0.01 versus both the p27 +/− and p27 −/− mice using the unpaired Students T test. The p27 +/− Lck-Bcl-2 mice were not significantly different than the P27 −/− Lck-Bcl-2 mice in this age group (P = 0.94).

The large increase in splenic T cells prompted us to examine thymocyte cellularity and proliferation. While we found large variations in thymic cellularity even within the same genotype, on average Bcl-2 expression reduced thymocyte cell number in p27 +/− mice while cell number was increased by Bcl-2 in p27 −/− mice (Figure 3A). We next examined the effect of p27 deficiency on the percentage of proliferating thymocytes (%S/G2/M). For this data, we found an age dependent effect of Bcl-2 on cell proliferation. In 4–6 week old mice, while Bcl-2 decreased the percentage of cycling cells in both p27 +/− and p27 −/− mice, the p27 −/− cohort had significantly higher percentage of cycling cells compared to the p27 +/− group (Figure 3B). However, in 7–12 week old mice, Bcl-2 inhibited thymocyte cell proliferation to the same extent in either p27 +/− or p27 −/− mice (Figure 3C).

### Bcl-2 delays cell cycle entry in p27−/− mice

**Figure 4:**
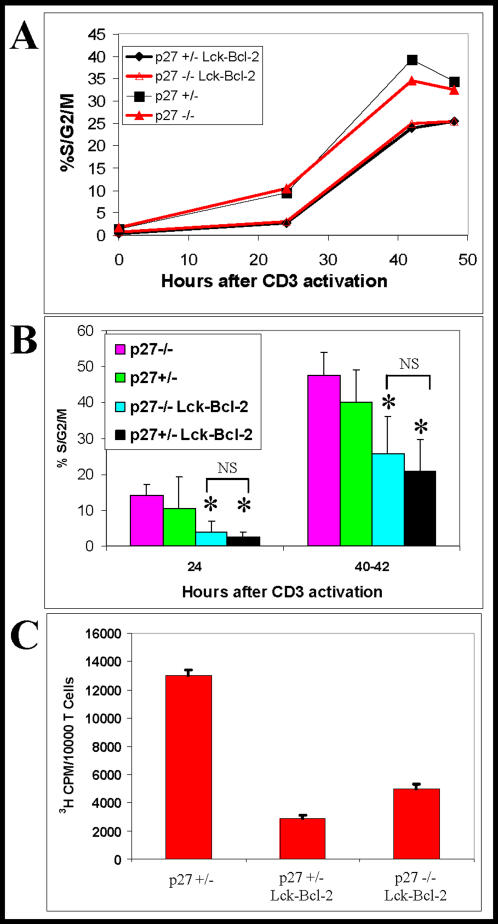
Bcl-2 retains anti-proliferative activity in p27 −/− mice. Splenic T cells were isolated, purified, and activated with immobilized anti-CD3 antibody as described in the Materials and Methods. A) At the time points indicated, T cells were harvested and analyzed for cell cycle using PI staining. The %S/G2/M at each time point is shown. This experiment is representative of at least three independent experiments. B) Shown is the %S/G2/M (mean±SD) cell cycle analysis at 24 and 40–42 hours following activation from multiple independent experiments from mice of the indicated genotypes. The differences between the Lck-Bcl-2 samples (p27 +/− vs p27 −/−) were not significant (NS). * P<0.01 versus the control p27 +/− and p27 −/− mice using the unpaired Students T test. C) Proliferation of T cells of the indicated genotypes was determined by ^3^H-thymidine uptake. Values represent Mean±SD of quintuplet samples. All three groups are significantly different from one another with a P value of <0.01 using the Students T test.

P27 has been proposed to be a critical mediator of the anti-proliferative function of Bcl-2 [29], [30]. To examine if p27 is required for delayed proliferation in Lck-Bcl-2 transgenic mice, purified splenic T cells from Lck-Bcl-2 p27−/− and control mice were activated with immobilized anti-CD3 antibody. A time course analysis demonstrated that Bcl-2 significantly delayed entry into the cell cycle (%S/G2/M). Surprisingly, the cell cycle delay by Bcl-2 was nearly equivalent in either p27 −/− or p27 +/− mice (Figure 4A). Examination of cell cycle by propidium iodide (PI) staining in multiple independent experiments showed that the percentage of cells that had entered the cell cycle was markedly reduced by Bcl-2 even in p27 −/− cells. This was true at either 24 or 40–42 hours (Figure 4B). When proliferation was measured by ^3^H-thymidine uptake, Bcl-2 effectively inhibited proliferation in p27 +/− and p27 −/− cells (Figure 4C).

### Delayed CDK2 phosphorylation in Bcl-2 T cells is independent of p27

**Figure 5:**
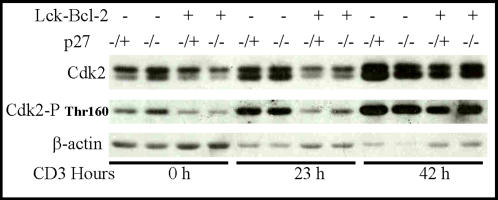
Bcl-2 inhibition of Cdk2 phosphorylation is independent of p27. T cells from mice of the indicated genotypes were purified and activated with immobilized anti-CD3 antibody prior to harvesting for immunoblot analysis at the time indicated. Immunoblots for total Cdk2, Phospho Thr-160, and Actin as a loading control are shown.

Previous results suggested that Bcl-2 delayed cell cycle correlates with delayed CDK2 activation [34]. This delay has been attributed at least in part to the elevation of p27 in cells expressing Bcl-2. We therefore examined the effect of Bcl-2 on Thr-160 phosphorylation of CDK2 in cells deficient in p27. Phosphorylation at this site has been tightly correlated with activity and is associated with a conformational change in the protein that can be detected by SDS-PAGE [35]. As expected Bcl-2 significantly delayed Thr-160 phosphorylation in p27 sufficient cells after CD3 activation. Surprisingly, a similar inhibition of CDK2 phosphorylation is detected even in p27 −/− T cells (Figure 5). For both p27 +/− and p27 −/− cells, Thr-160 phosphorylation is largely normalized 42 h after CD3 activation showing the delay is transient. These results demonstrate that the delay in CDK2 phosphorylation by Bcl-2 is independent of p27 in this model.

### Cyclin D2 and D3 expression is decreased in Lck-Bcl-2 transgenic mice

**Figure 6:**
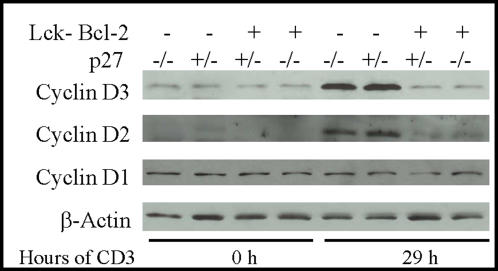
Bcl-2 delays the upregulation of Cyclins D2 and D3 independent of p27. T cells from mice of the indicated genotypes were purified and activated with immobilized anti-CD3 antibody prior to harvesting for immunoblot analysis at the time indicated. Immunoblots for total Cyclin D1, Cyclin D2, Cyclin D3, and Actin as a loading control are shown.

The progression from G0 to G1 requires the activation of D cyclins prior to the phosphorylation/activation of CDK2. Since the delay in CDK2 phosphorylation was independent of p27, the levels of D type cyclins in Lck-Bcl-2 transgenic mice prior to and after activation with CD3 were determined. Interestingly, there was no change in cyclin D1 levels in T cells following anti-CD3 activation (Figure 6). However, both Cyclin D2 and D3 are markedly increased 29 h after activation with anti-CD3 in the control cells not expressing Bcl-2. In contrast, T cells expressing Bcl-2 did not show an increase in cyclin D2 and D3 29 h after activation. This was true for both p27 +/− and p27 −/− Bcl-2 expressing cells.

### Bcl-2 and p27 deficiency cooperate in lymphoma formation

**Figure 7:**
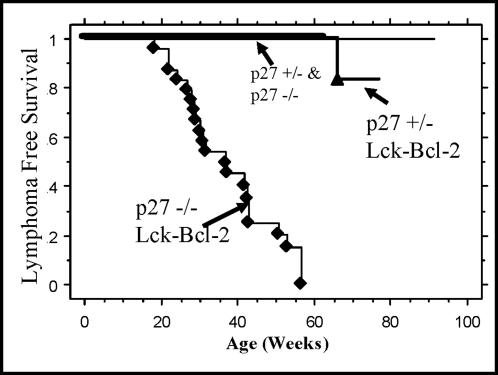
Bcl-2 synergizes with p27 deficiency to rapidly promote lymphoma formation. Mice of the indicated genotype were followed for thymic tumor free survival as described in the Materials and Methods. Kaplan-Meier lymphoma free survival analysis of mice of the indicated genotypes is shown. Lymphoma formation in p27 −/− Lck-Bcl-2 mice was significantly (P<0.01) accelerated compared to all three of the control groups (p27 +/−, p27 −/−, and p27 +/− Lck-Bcl-2). The other three groups were not significantly different from one another with regards to survival due to thymic tumors. As previously reported, a subset of p27 −/− mice did succumb to pituitary adenomas but these animals had no evidence of thymic lymphomas and were censored from this analysis of lymphoma formation (data not shown).

Since previous studies implicated p27 in cell cycle regulation by Bcl-2 [30] and our preliminary studies demonstrate dramatic lymphoid hyperplasia and an increase in thymocyte proliferation in young p27 −/− Lck-Bcl-2 mice, lymphoma formation was examined in these animals. Previous studies found that lymphoma formation is observed in Lck-Bcl-2 transgenic mice but significant penetrance is not observed until after 1 year of age [36]. Similarly, lymphoma formation is infrequent in p27 −/− mice in the first year of life [37]. In this study, p27 −/− Lck-Bcl-2 and control groups were monitored weekly for evidence of tumors. P27 −/− Lck-Bcl-2 mice were highly susceptible to thymic lymphomas with complete penetrance by 60 weeks of age. Consistent with previous reports, lymphomas were infrequently detected in p27 +/−, p27 −/−, or Lck-Bcl-2 transgenic mice during this time period (Figure 7).

### P27 −/− Lck-Bcl-2 thymic lymphomas are lymphoblastic and involve multiple organs

**Figure 8:**
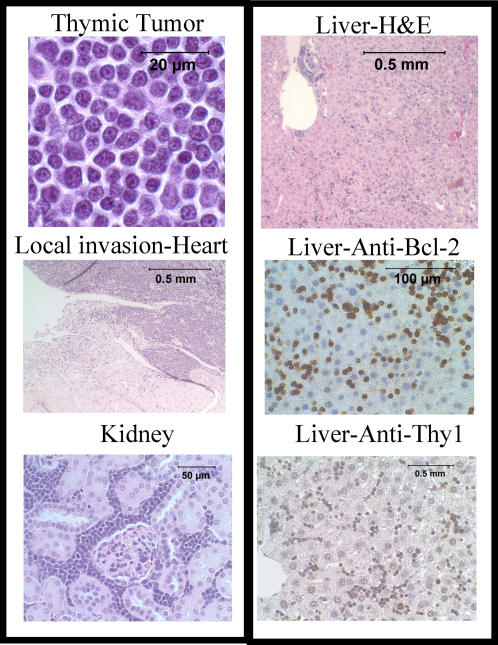
Tumors from p27 −/− Lck-Bcl-2 mice are aggressive T cell lymphoblastic lymphomas. Histological analysis of the thoracic tumors from the p27 −/− Lck-Bcl-2 mice is shown as indicated. H&E staining shows a characteristic lymphoblastic lymphoma (Top left) that were frequently locally invasive (Heart) and involved distant organs such as the kidney and liver. Immunohistochemistry staining of the liver confirms the tumors are of T cell origin (Thy1 positive) and retain expression of human BCL-2.

Necropsies of the p27 −/− Lck-Bcl-2 animals generally showed a massively enlarged thoracic tumor that in all cases appeared to arise within the thymus. Most animals were sacrificed due to respiratory compromise/labored breathing from the tumor. Histological examination of H&E stained tumors showed the tumor contained lymphoblastic cells (Figure 8). Histological examination of the tumors demonstrated locally invasive tumors that frequently involved the kidneys and liver (Figure 8). Immunohistochemical staining showed the tumor cells were Thy1 positive and retained expression of human Bcl-2 (Figure 8). These results confirm that the thoracic tumors are aggressive T cell thymic lymphomas.

### P27 deficiency fails to promote lymphoma development in Lck-Bax38/1mice

**Figure 9:**
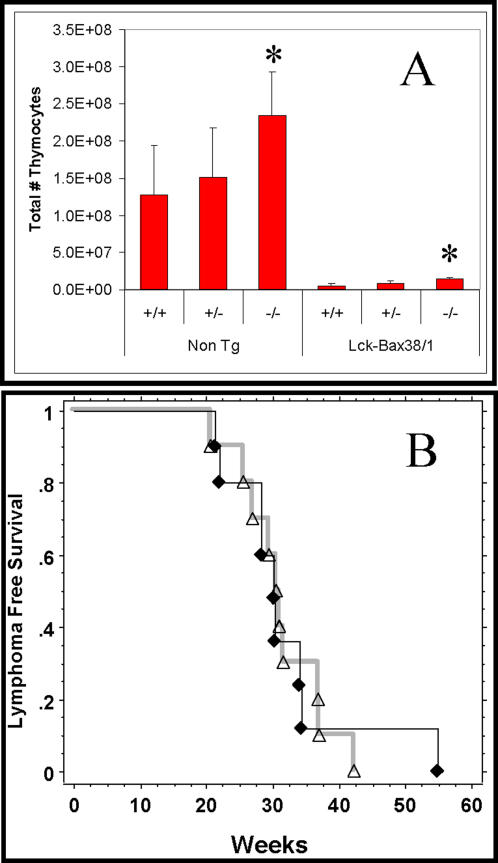
p27 deficiency does not accelerate lymphoma formation in Lck-Bax38/1 mice. A) Thymic cellularity from young mice of the indicated genotypes is shown. Data represent the Mean±SD of at least four animals from each group. In all cases, Lck-Bax38/1 mice had significantly reduced cellularity compared to the three transgene negative control groups shown on the left. *P<0.05 vs the respective p27 +/+ and p27 +/− controls. B) Kaplan-Meier analysis for lymphoma free survival comparing p27 −/− Lck-Bax38/1 (♦) and p27 +/− Lck-Bax38/1 (Δ) mice is shown.

In contrast to Bcl-2, Bax has been shown to have pro-apoptotic function. Despite this pro-apoptotic activity, expression of Bax in thymocytes accelerates T cell lymphoma development [18], [38]. Given the cooperation between p27 deficiency and Bcl-2, the effect of p27 deficiency on lymphoma formation in Lck-Bax38/1 transgenic mice was determined. In young mice, increased Bax expression results in dramatic thymic lymphopenia concordant with previous findings [19] which was modestly increased in p27 −/− mice. This was true for both control and Lck-Bax38/1 transgenic mice (Figure 9A). However, in contrast to Lck-Bcl-2 mice, p27 deficiency had no affect on the development of lymphoma formation in Lck-Bax38/1 transgenic mice (Figure 9B).

## Discussion

Numerous studies in both humans and mice have shown paradoxical effects of Bcl-2 on tumor formation. Several studies have attributed these paradoxical effects to the anti-proliferative activity of Bcl-2. Bcl-2 mediated inhibition of proliferation has been tightly correlated with the anti-apoptotic function of Bcl-2 and to increased levels of p27 [28]–[30]. In fact, in one model Bcl-2 expression was unable to inhibit T cell proliferation in p27 deficient cells [30]. These studies prompted us to determine if p27 is an important tumor suppressor downstream of Bcl-2. In this study, we hypothesized that p27 deficiency would cooperate with Bcl-2 in tumor formation. Consistent with this hypothesis, lymphoma formation was dramatically accelerated by combining the loss of p27 with the expression of Bcl-2.

CKI's inhibitors include both the Cip/Kip [39] and INK4 [40] families. For the INK family, p16 has been clearly shown to be a tumor suppressor [40], [41]. For the members of the Cip/Kip family, their roles as bonafide tumor suppressors are not as well established. P21 was one of the first known targets of p53 and as such was proposed to act as a tumor suppressor. Surprisingly, p21 deficient mice failed to develop tumors at a significant rate [42], [43]. Similarly, p27 deficient mice demonstrate multiple defects in growth control but are not particularly prone to tumor formation [37], [44]. Though some of the mice develop pituitary adenomas, spontaneous aggressive malignancies are rare. However, p27 deficient mice are prone to develop aggressive malignancies when combined with a deletion in tumor suppressor genes such as p53 or INK4a/ARF, or when challenged with mutagens such as X-irradiation or N-ethyl-N-nitrosourea (ENU) [45]–[47]. Moreover, lymphoma formation was rapidly accelerated in p27 deficient mice expressing the dominant oncogenes myc (Eμ-myc mice) or cyclin E [48], [49]. Together, these findings demonstrate that the absence of p27 by itself is not predominantly oncogenic; however, p27 deficiency can sensitize mice to other oncogenic insults. The study described here extends these studies and demonstrates that p27 is an important tumor suppressor in the context of Bcl-2 expression.

In contrast to the result with Bcl-2, the absence of p27 had no impact on lymphoma formation in mice expressing pro-apoptotic Bax. The data clearly show that complete elimination of p27 had no additive effect on tumor formation and only a modest increase in thymic cellularity in young Lck-Bax38/1 mice. These results support the idea that Bax and Bcl-2 mediate tumor formation by distinct pathways, consistent with their opposing function in regulating cell death. Previous studies have shown that Bcl-2 expression maintains genome stability by abrogating the selective pressure(s) needed to lose p53 in experimental tumor models [50], [51]. However, the Bcl-2 family has paradoxical effects on lymphoma formation and chromosomal instability even in p53 −/− mice [18], [19]. These data demonstrate the paradoxical effects of the Bcl-2 family are in some cases independent of p53 and suggest that the effects of Bcl-2 on tumor formation are not singularly dependent on whether the tumors retain or lose p53. These studies in mice are consistent with a recent study of human breast cancer that showed Bcl-2 expression provides a favorable prognosis independent of several risk factors including the Nottingham Prognostic Index and was a more important prognostic factor than p53 status [52].

The ability of Bcl-2 to retain its cell cycle inhibitory affect on splenic T cells in the absence of p27 was surprising. A previous report had found that Bcl-2 expression in T cells was unable to inhibit ^3^H-thymidine uptake in p27 deficient cells [30]. This is in contrast to our findings that Bcl-2 effectively inhibits splenic T cell proliferation as measured by cell cycle analysis of PI stained nuclei or ^3^H-thymidine uptake in p27 deficient cells. While the explanation for this discrepancy is not clear, one possibility may be that the studies employed different transgenic mouse models. Our study utilized the Lck promoter and the study by Vairo et. al. used a Eμ-promoter. It is possible that Bcl-2 expression is higher in the Lck model and the singularly important role of p27 is apparent at lower levels of Bcl-2 expression. In any case, we did find a role for p27 in a developmentally dependent manner. We demonstrated that Bcl-2 more effectively inhibited thymocyte proliferation in p27 +/− mice compared to p27 −/− mice in mice between 4 and 6 weeks of age. These differences between mice with and without p27 were not observed in older mice between 7 and 12 weeks of age. These results suggest that the effects of p27 in this model are age dependent and as the animal ages, the extent of T cell hyperplasia becomes more pronounced. The cells are then reduced in size and p27 independent pathways are able to inhibit T cell proliferation. Our studies suggest that delayed up regulation of Cyclin D2 and Cyclin D3 may mediate the p27 independent cell cycle inhibition by Bcl-2.

The results described confirm and extend our previous report in which inhibition of T cell proliferation by Bcl-2 was dependent upon the age of the mouse, the extent of T cell hyperplasia and the reduction in T cell size [29]. In our working model, we propose that a decrease in a survival/proliferative signal results in a reduction in T cell size and a delay in cell proliferation. For T cells, the *in vivo* survival/proliferative signal may involve interactions between the T Cell Receptor (TCR) on the T cells with the Major Histocompatibility Complex (MHC) on antigen presenting cells (APCs) [53]. When Bcl-2 is expressed, the selective reduction of T cell death leads to an increase in the ratio of T Cells to APC's in an age dependent manner. This altered ratio decreases the TCR/MHC interactions, results in the reduction in T cell size and delayed proliferation. Our studies show the primary function of p27 is to regulate the extent of T cell hyperplasia while inhibition of T cell size and cell cycle remains intact even in p27 deficient mice.

The studies here demonstrate that Bcl-2 significantly delays the increase in expression of both Cyclin D2 and Cyclin D3 in a manner that is independent of p27. This delay correlates with a delay in Cdk2 phosphorylation which has been tightly linked to exit from G1. This data provide yet another mechanism by which Bcl-2 may inhibit cell cycle in addition to the p27 and p130 pathways [30]. The studies here have not addressed the mechanism by which Bcl-2 delays the up-regulation of Cyclin D2 and D3. However, other studies show the levels of the Cyclin D family are controlled by either protein stability [54] or mRNA levels [55]. In summary, these data demonstrate a complex relationship between the Bcl-2 family, cellular proliferation, and oncogenesis and demonstrate that p27 up-regulation is not singularly important in the Bcl-2 mediated proliferative delay observed in T cells. Nonetheless, the results indicate that p27 plays a critical role in tissue homeostasis and in tumor development in the context of Bcl-2 expression.

## Materials and Methods

### Transgenic mice

Lck-Bcl-2 and Lck-Bax38/1 were previously described and were genotyped by PCR [33], [38]. Lck-Bax38/1 are mice that harbor two independent transgenic Bax constructs as previously described [18]. P27 deficient mice [45] were obtained from Jackson Labs (Stock #003122) and were genotyped by PCR as recommended by Jackson Labs.

### Tumor development studies

All mice were maintained in the animal facility at the University of Iowa under an approved protocol (ACURF #0310191). Mice of the appropriate genotypes were mated to obtain animals for the tumor development studies. Littermate controls or controls from the same mating pairs were used for all these studies. When possible, p27 −/− mice were used for matings to provide the highest percentage of transgene positive-p27 deficient mice. Therefore, p27 +/− mice were used as controls. Upon entry into the tumor development study, animals were examined weekly for signs of illness or malignancy. Sick animals were monitored more frequently and euthanized when necessary to prevent unnecessary suffering. Control animals were monitored for at least one year and then killed for necropsy. These animals did not show any evidence of lymphoma and were all censored from the analysis of tumor free survival. When possible, necropsies were performed on dead animals to determine if the animals had gross evidence of lymphoma. Tumors were then confirmed by fixation with formalin and histological examination after H&E staining. All mice were maintained in the animal facility at the University of Iowa (Iowa City, IA). Statistical analysis was performed with the StatView Program (SAS Institute Inc.) using Kaplan-Meier cumulative survival and the Logrank (Mantel-Cox) test to determine if differences in survival were significant between each of the indicated groups.

### Cell preparation, analysis and CD3 activation

Single cell suspensions from either the spleen or thymus were prepared by dispersing the organ between two glass slides in PBS. Red blood cells were removed by 5-minute incubation in hypotonic lysis buffer (0.83% NH_4_Cl, 10 mM Tris/pH 7.2). The viable cell counts for both the thymus and the spleen were determined using a hemocytometer and trypan blue exclusion or using Viacount reagent with the Guava flow cytometer (Guava Technologies, Hayward, California, USA) as previously described [56]. Splenic T cells were quantified by staining the cells with anti-CD3 (eBioscience #16-0031) and anti-B220 (eBioscience #12-0452) antibodies and analyzed by flow cytometry as previously described [33]. For cell size determination, the mean fluorescence intensity of the forward scatter parameter (FSC) was determined for the CD3 positive cells. At least 5 mice between 5 and 12 weeks of age was recorded for each of the 4 groups. Differences between groups were determined by the t-Test assuming unequal variance (within Microsoft excel). Total splenic T cells were calculated by multiplying the %CD3-positive by the total number of splenic cells.

Splenic T cells were purified using reagents and protocols from Stem Cell Technologies (Product #13051) as previously described [29], [57]. Cell purity was generally greater than 95% based on CD3-positive staining. Purified T cells (0.5 to 1.0 million cells/ml) were seeded onto either 96 well plates (0.2 ml for cell cycle studies) or 6 well plates (5–6 ml for immunoblots) that were pre-coated with saturating amounts of anti-CD3 antibody (145-2C11 eBioscience #16-0031-85). Cells were cultured in RPMI-1640 supplemented with 10% FBS, 0.1 mM 2-mercaptoethanol, 200 mM glutamine, and penicillin/streptomycin. At various times following activation, cells were harvested and prepared for analysis. Cell cycle PI staining and ^3^H-thymidine analysis was performed as previously described [29]. Cell viability was performed in duplicate using the Guava Flow cytometer and the ViaCount reagent as previously described [56]. For immunoblot analysis, cells were harvested, lysates prepared, and blots performed as previously described [29]. Antibodies used for immunoblot analysis were purchased as follows: pCDK2-Thr-160 (Cell Signaling Technology #2561S); CDK2 (Santa Cruz #SC-163); Actin (Sigma #A4700); Cyclin D1 (Sigma #C7464); Cyclin D2 (Sigma #C7339); cyclin D3 (BD Pharminigen #610279).
